# Marginal Zone B Cells in Neonatal Rats Express Intermediate Levels of CD90 (Thy-1)

**DOI:** 10.1080/10446670310001593488

**Published:** 2002-12

**Authors:** Peter M. Dammers, Monique E. Lodewijk, André Zandvoort, Frans G. M. Kroese

**Affiliations:** 1 Department of Cell Biology Immunology Section University of Groningen A. Deusinglaan 1, AV Groningen NL-9713 The Netherlands; 2 Department of Pathology and Laboratory Medicine University Hospital Groningen P.O. Box 30.001 RB Groningen NL-9700 The Netherlands

## Abstract

Here we show that marginal zone (MZ)-B cells in rats can already be detected in neonatal spleen from two days after birth. At this time point, morphologically distinct MZs are not present yet and the vast majority of B cells in spleen are located in a concentric area surrounding the T cell zones (PALS). Before MZs are obviously detectable in spleen (14 days after birth), MZ-B cells seem to be enriched at the outer zones of the concentric B cell areas. Similar to adult rats, neonatal MZ-B cells are intermediate-sized cells that express high levels of surface (s)IgM and HIS57 antigen, and low levels of sIgD and CD45R (HIS24). We show here, however, that in contrast to adult MZ-B cells, MZ-B cells (and also recirculating follicular (RF)-B cells) in neonatal rats express higher levels of CD90 (Thy-1). In adult rats, expression of CD90 on the B cell lineage is confined to immature B cells. We speculate that the expression of CD90 on neonatal MZ-B cells may have implications for their responsiveness to polysaccharide (T cell-independent type 2) antigens.


*Developmental Immunology*, December 2002 Vol. 9 (4), pp. 187–195

Taylor & Francis healthsciences

### Marginal Zone B Cells in Neonatal Rats Express Intermediate Levels of CD90 (Thy-1)*

PETER M. DAMMERS[a], MONIQUE E. LODEWIJK[b], ANDRÉ ZANDVOORT[b] and FRANS G.M. KROESE[a,†]

[a]*Department of Cell Biology, Immunology Section, University of Groningen, A. Deusinglaan 1, NL-9713 AV Groningen, The Netherlands;* [b]*Department of Pathology and Laboratory Medicine, University Hospital Groningen, P.O. Box 30.001, NL-9700 RB Groningen, The Netherlands*

Here we show that marginal zone (MZ)-B cells in rats can already be detected in neonatal spleen from two days after birth. At this time point, morphologically distinct MZs are not present yet and the vast majority of B cells in spleen are located in a concentric area surrounding the T cell zones (PALS). Before MZs are obviously detectable in spleen (14 days after birth), MZ-B cells seem to be enriched at the outer zones of the concentric B cell areas. Similar to adult rats, neonatal MZ-B cells are intermediate-sized cells that express high levels of surface (s)IgM and HIS57 antigen, and low levels of sIgD and CD45R (HIS24). We show here, however, that in contrast to adult MZ-B cells, MZ-B cells (and also recirculating follicular (RF)-B cells) in neonatal rats express higher levels of CD90 (Thy-1). In adult rats, expression of CD90 on the B cell lineage is confined to immature B cells. We speculate that the expression of CD90 on neonatal MZ-B cells may have implications for their responsiveness to polysaccharide (T cell-independent type 2) antigens.

*Keywords*: B cell; Marginal zone; Ontogeny; Spleen; Rat

### INTRODUCTION

In mammals, splenic marginal zone (MZ)-B cells are functionally implicated in the antibody (Ab) response against bloodborn micro-organisms, especially against encapsulated bacteria like *Streptococcus pneumoniae*, *Neisseria meningitidis* and *Haemophilus influenzae* (MacLennan and Liu, 1991; Spencer *et al.*, 1998). The capsules of these bacteria consist of polysaccharides which characteristically elicit T cell-independent type 2 (TI-2) Ab responses upon infection (Mosier and Subbarao, 1982; Mond *et al.*, 1995). Experiments in mice revealed that survival from infection with encapsulated bacteria strongly correlates with the ability of the host to mount a rapid anti-polysaccharide Ab response (Peltola *et al.*, 1977; Feeney *et al.*, 1996; Guo *et al.*, 1997; Benedict and Kearney, 1999).

In humans, responsiveness to TI-2 antigens does not develop until several months after birth and does not reach adult levels until after 5 years of age (Pabst and Kreth, 1980; Rijkers *et al.*, 1998). This might be one of the reasons that explains the high incidence of death resulting from infection with encapsulated bacteria in young children. A similar phenomenon is observed in mice, where responsiveness to TI-2 antigens also appears to develop relatively late in ontogeny (Mosier *et al.*, 1977). In addition to the immaturity of macrophages and the rather low serum levels of many components of the complement pathway (especially components of the alternative pathway) (Pabst and Kreth, 1980), the weak TI-2 antibody responses in neonates and young children may well result from the absence and/or immaturity of the splenic MZ-B cells at childhood (Timens *et al.*, 1989; Rijkers *et al.*, 1998; Zandvoort *et al.*, 2001a). In both human and rodent spleen, B cell follicles and MZs have not yet developed at birth (Friedberg and Weissman, 1974; Dijkstra and Dopp, 1983; Kroese *et al.*, 1987; Timens *et al.*, 1989). Dijkstra and Dopp, 1983 showed by immunohistology using polyclonal Abs that MZ-B cells in rats can be phenotypically distinghuished in the spleen as early as 6 days after birth. At this time, B cells are found in spleen in a concentric ring surrounding the periarteriolar lymphoid shealth (PALS). B cells located at the outermost border of these concentric areas, exhibit a strong membrane staining for IgM. Dijkstra and Dopp, 1983 suggested that these $IgM^{high}$ cells likely represent MZ-B cells.

Several studies have provided evidence that MZ-B cells have a mixed origin and may contain "naive" (non-memory) B cells as well as memory B cells. In human adults, most MZ-B cells are memory B cells, as is concluded from the somatic mutations found in

*Presented at the Proceedings of the 4th Germinal Center Conference, June 2002, Groningen, The Netherlands.

†Corresponding author. Tel.: +31-050-3632534. Fax: +31-050-3632512. E-mail: f.g.m.kroese@med.rug.nl

ISSN 1044-6672 print © 2002 Taylor & Francis Ltd
DOI: 10.1080/10446670310001593488

the immunoglobulin (Ig) heavy (H) chain variable region ($V_H$) genes of human MZ-B cells (Dunn-Walters *et al.*, 1995; Tierens *et al.*, 1999). In addition, most MZ-B cells in man express the memory B cell marker CD27 (Tangye *et al.*, 1998). In adult mice and rats, however, most MZ-B cells express germline encoded Ig $V_H$ genes, indicating that the vast majority of MZ-B cells in the spleen of these species consist of non-memory B cells (Makowska *et al.*, 1999; Dammers *et al.*, 2000). Similarly, very few memory B cells are found in human spleens at birth, as is illustrated by the fact that only very few MZ-B cells express CD27 at that time (Zandvoort *et al.*, 2001a). We, and others demonstrated that the non-memory MZ-B cells can arise directly from mature RF-B cells without the requirement for proliferation (MacLennan and Liu, 1991; Dammers *et al.*, 1999). In addition, we recently showed that MZ-B cells in normal adult rats display a selected Ig repertoire (Dammers *et al.*, 2000), which is most likely the result of positive selection constrains (Martin and Kearney, 2000). If the MZ-B cell selection constrains are different during ontogeny, this might well be a possible explanation for the lack of responsiveness to TI-2 antigens in early mammalian life, because it would lead to a different and perhaps for TI-2 antigens less adapted Ig repertoire.

To address issues related to the selection process of MZ-B cells, we first studied the neonatal development of MZ-B cells in rats, in terms of phenotype and immunohistological localization *in vivo*. Here, we show that MZ-B cells can already be detected by flow cytometry and immunohistology by two days after birth. Furthermore, for at least the first three weeks after birth, neonatal MZ-B cells were found to express intermediate levels of the "immature B cell marker" CD90.

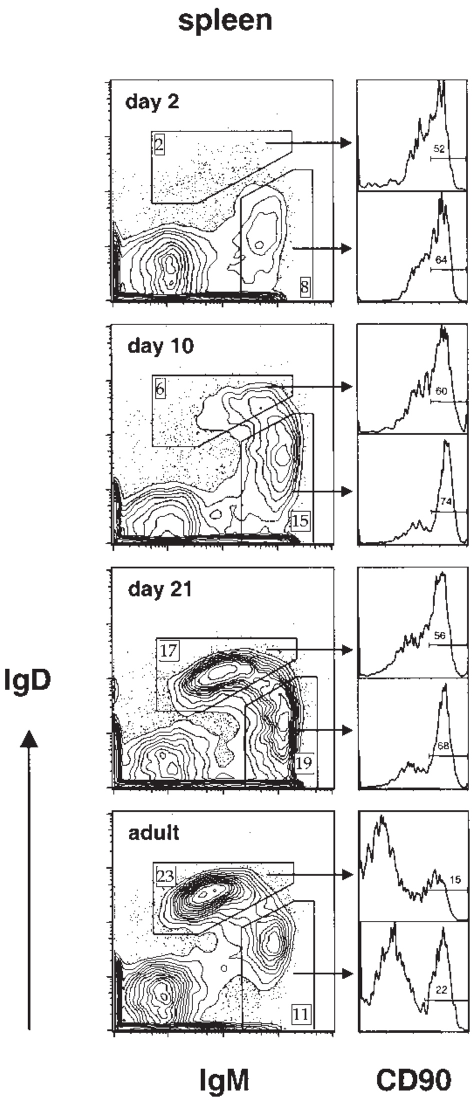

FIGURE 1 Flow cytometry analysis of neonatal rat spleen stained for IgM, IgD and CD90. Spleens were taken 2, 10 and 21 days after birth and from adult rats (5-months-old). In adult rat spleen, the sIgM$^{high}$sIgD$^{low}$ B cell subset consists of NF-B cells (CD90$^{high}$) and MZ-B cells (CD90$^{neg}$), and the sIgM$^{low}$sIgD$^{high}$ B cell subset consists of ERF-B cells (CD90$^{high}$) and RF-B cells (CD90$^{neg}$). Note the presence of cells that express intermediate levels of CD90 and the virtual absence of CD90$^{neg}$ cells within the IgM/IgD-defined B cell subsets during neonatal spleen development. Contour plots (5% probability) were generated on the basis of 40,000 events, after gating for lymphoid cells on the basis of forward-sideward scatter profiles. Numbers represent percentages within the lymphoid gate, except for CD90 where numbers represent percentage of cells within the respective IgM/IgD gate.

### RESULTS

### Flow Cytometry Analysis Reveals the presence of Splenic MZ-B Cells already Two Days after Birth

In order to study MZ-B cell development in neonatal rats, spleen cell-suspensions were prepared from 2-, 10-, 14- and 21-days old animals and analyzed by three-color flow cytometry. MZ-B cells can be detected in adult spleen by virtue of their unique phenotype: MZ-B cells are sIgM$^{high}$sIgD$^{low}$CD45R$^{low}$CD90$^{neg}$ and express high levels of an antigen recognized by the newly developed mAb HIS57 (Dammers *et al.*, 1999). In adult rat spleen, staining for IgM and IgD reveals the presence of two B cell subsets: sIgM$^{low}$sIgD$^{high}$ and sIgM$^{high}$sIgD$^{low}$ B cells, both of which can be further subdivided by virtue of CD90 expression levels. The sIgM$^{low}$sIgD$^{high}$ B cell subset comprise early recirculating follicular (ERF)-B cells (CD90$^{high}$) and recirculating follicular (RF)-B cells (CD90$^{neg}$), whereas the sIgM$^{high}$sIgD$^{low}$ B cell subset contains newly formed (NF)-B cells (CD90$^{high}$) and MZ-B cells (CD90$^{neg}$) (Kroese *et al.*, 1995). Figure 1 shows that sIgM$^{high}$sIgD$^{low}$ B cells predominate in spleen during the first three weeks after birth. Two days after birth, the percentage of sIgM$^{high}$sIgD$^{low}$ B cells is about four times higher than the percentage of sIgM$^{low}$sIgD$^{high}$ B cells, whereas in adult rats there are generally about two times more sIgM$^{low}$sIgD$^{high}$ B cells than sIgM$^{high}$sIgD$^{low}$ B cells. Figure 1 also shows that, in contrast to adult animals, all IgM/IgD-defined neonatal B cells express intermediate or high levels of CD90, whereas CD90$^{neg}$ cells are virtually absent. Two days after birth, about 50%

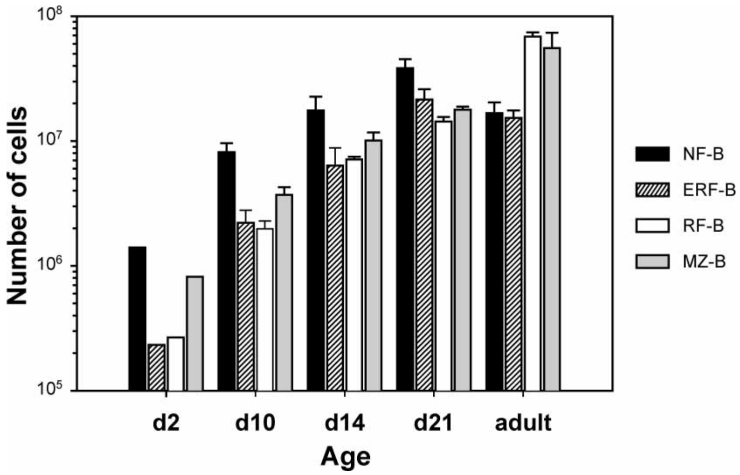

FIGURE 2 Absolute numbers of NF-B, ERF-B, RF-B and MZ-B cells in spleen of neonatal PVG rats (2, 10, 14 and 21 days after birth) and adult rats (5-months-old). B cell subsets were determined by means of three-color flow cytometry using anti-IgM, anti-IgD and anti-CD90 staining (see text). For neonatal animals, RF-B and MZ-B cells were defined as $CD90^{int/neg}$ cells (within the respective IgM/IgD gate). For each time point, the absolute number of cells per subset is the average of three animals, except for the adult ($n = 4$). Error bars represent the standard deviation (SD).

of the splenic $sIgM^{high}sIgD^{low}$ B cells express high levels of CD90 and therefore represent NF-B cells (Fig. 1). As shown in Fig. 2, the absolute number of NF-B cells in neonatal spleen already reaches adult levels by 14 days after birth. At weaning (day 21), the number of NF-B cells in spleen exceeds that of adult rats by two-fold. The other $sIgM^{high}sIgD^{low}$ neonatal B cells express intermediate levels of CD90, a phenotype that is not seen in adult animals. These neonatal $sIgM^{high}sIgD^{low}CD90^{int}$ cells are most probably MZ-B cells. MZ-B cells in adult rats express low levels of CD45R (HIS24) and high levels of HIS57 antigen. As we show in Fig. 3, cells with such a phenotype are also seen in neonatal spleens. Already two days after birth, low numbers of $sIgM^{high}HIS57^{high}$ and $sIgM^{high}CD45R^{low}$ MZ-B cells can be distinguished. Consistent with a MZ-B cell phenotype, these cells are relatively large, as revealed by their higher forward-scatter profile, compared to other B cells in spleen. Furthermore, we show that neonatal MZ-B cells defined by staining for sIgM in combination with CD45R (HIS24) or HIS57 antigen, express intermediate levels of CD90. The CD90 expression levels are the highest on MZ-B cells taken from spleens two days after birth and these levels gradually diminish with age. Absolute numbers of MZ-B cells increase more than 10-fold during the first three weeks of life (Fig. 2).

During the first three weeks after birth, the vast majority of the $sIgM^{low}sIgD^{high}$ B cells express relatively high levels of CD90 and likely represent ERF-B cells (Fig. 1). In adult rats, ERF-B cells constitute only a relatively small proportion of $sIgM^{low}sIgD^{high}$ B cells. The other B cells belonging to the $sIgM^{low}sIgD^{high}$ subset, express intermediate levels of CD90. The expression levels of CD90 on these cells diminish gradually over time. We speculate here that the $IgM^{low}sIgD^{high}CD90^{int}$ B cells may well represent RF-B cells that express some CD90, similar to MZ-B cells. Indeed, numbers of $sIgM^{low}sIgD^{high}CD90^{int}$ B cells increase with age (Fig. 2), which can be expected for the expanding RF-B cell pool.

Figure 4 shows that two days after birth, appreciable numbers of $sIgM^{neg}CD90^{high}CD24^{high}$ cells can be detected in rat spleen. In adult bone marrow these cells represent pro/pre-B cells (Hermans *et al.*, 1997). This observation indicates that, in addition to bone marrow, B lymphopoiesis also takes place in the neonatal spleen. The pro/pre-B cells in spleen probably contribute to a rapid generation of NF-B cells only during the first week of life, because splenic pro/pre-B cells are virtually absent by 10 days of age.

### Immunohistochemical Staining Reveals the presence of MZ-B Cells in Neonatal Animals

In order to study the *in situ* development of MZ and MZ-B cells in neonatal rat spleen, we stained serial frozen section of 2-, 10-, 14- and 21-days-old spleen for either IgM, IgD, Igκ, $TCR_{\alpha\beta}$, CD45R (HIS24), CD90 or HIS57 antigen. Two days after birth, small numbers of $TCR^{+}_{\alpha\beta}$ T cells are found in spleen which are predominantly located in an area around the central arterioles (PALS). At this age, most $IgM^{+}$ B cells reside in a distinct concentric area surrounding the PALS (Fig. 5). B cells located at the outermost border of these concentric areas exhibit more (membranous) IgM staining compared to B cells of the inner part. In addition to B cells in these concentric areas, small numbers of $IgM^{+}$ B cells and $IgM^{+}$ plasma cells can also be detected scattered throughout the red pulp. Two days after birth, the vast majority of $IgM^{+}$ B cells in spleen seem to express Igκ light-chains, since the number of $Ig\kappa^{+}$ and $IgM^{+}$ cells as well as the staining patterns for either IgM and Igκ are very similar. Figure 5 shows that only a fraction of the B cells in the concentric areas surrounding the PALS express IgD. A similar fraction also stains for CD45R

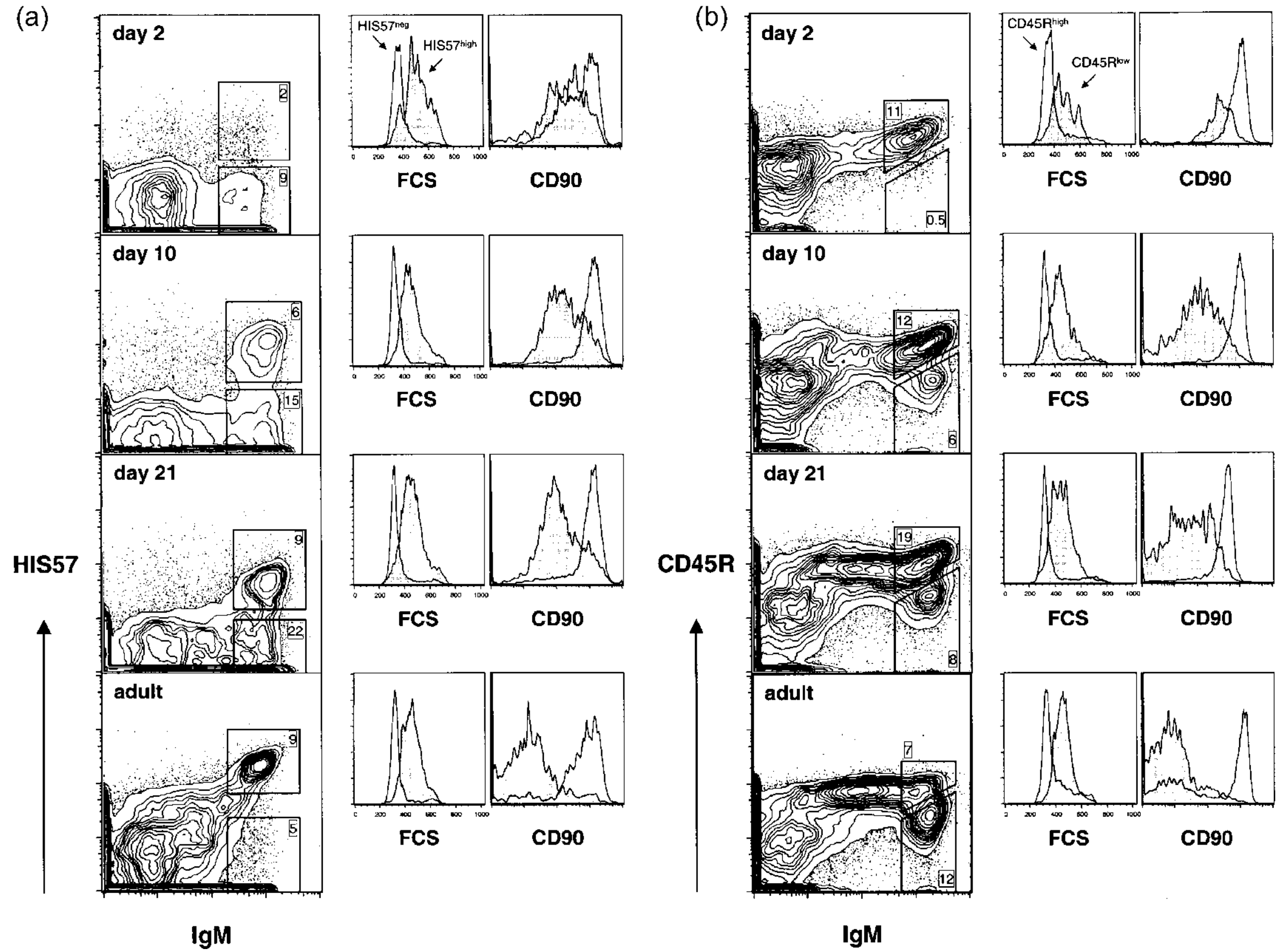

FIGURE 3 Three-color flow cytometry analysis of neonatal (2, 10 and 21 days after birth) and adult rat spleen (5-months-old). Figures show the forward scatter profiles (FCS) and CD90 expression levels on MZ-B cells (gray fill color), defined as $sIgM^{high}HIS57^{high}$ (a) and $sIgM^{high}CD45R^{low}$ (b) cells. For comparison, FCS and CD90 expression is also depicted for NF-B cells, defined as $sIgM^{high}HIS57^{neg}$ (a) and $sIgM^{high}CD45R^{high}$ (b) cells (white fill color). Contour plots (5% probability) were generated on the basis of 40,000 events and after gating for lymphoid cells using forward–sideward scatter profile. Numbers represent percentages within the lymphoid gate.

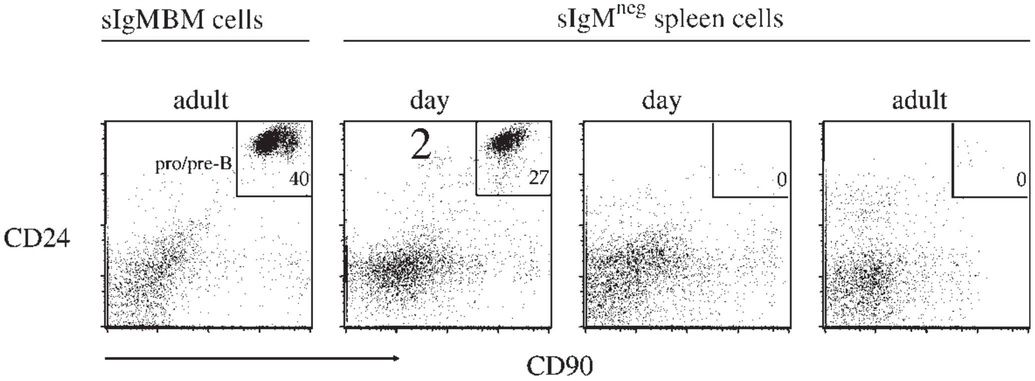

FIGURE 4 Flow cytometry analysis showing the presence of pro/pre-B cells in two-days-old rat spleen. Neonatal (day 2 and 10) and adult (5-months-old) spleen cells as well as adult bone marrow cells were stained for IgM, CD90 and CD24. Shown is the CD90 vs. CD24 expression pattern for sIgM$^{neg}$ cells. Dotplots were established on the basis of 40,000 events after gating for lymphoid cells using forward–sideward scatter profiles. Numbers represent percentages within the lymphoid gate. Note the presence of appreciable numbers of CD90$^{high}$CD24$^{high}$ cells (pro/pre-B cells) among the sIgM$^{neg}$ spleen cells two days after birth.

(HIS24) (data not shown). IgD$^{+}$ and CD45R$^{+}$ (HIS24) cells are enriched at the inner part of the concentric areas, and may well represent follicular type B cells (ERF-B and RF-B). Also in the red pulp some scattered CD45R$^{+}$ (HIS24) cells are found (approximately in equal numbers as IgM$^{+}$ cells), whereas only limited numbers of IgD$^{+}$ cells are present in this area. In addition to numerous cells in the red pulp, virtually all B cells in the concentric areas, stain for CD90. HIS57 staining was not observed in two-days-old spleen (data not shown).

While at day 10 after birth most B cells are still confined to the concentric areas surrounding the PALS, some initial

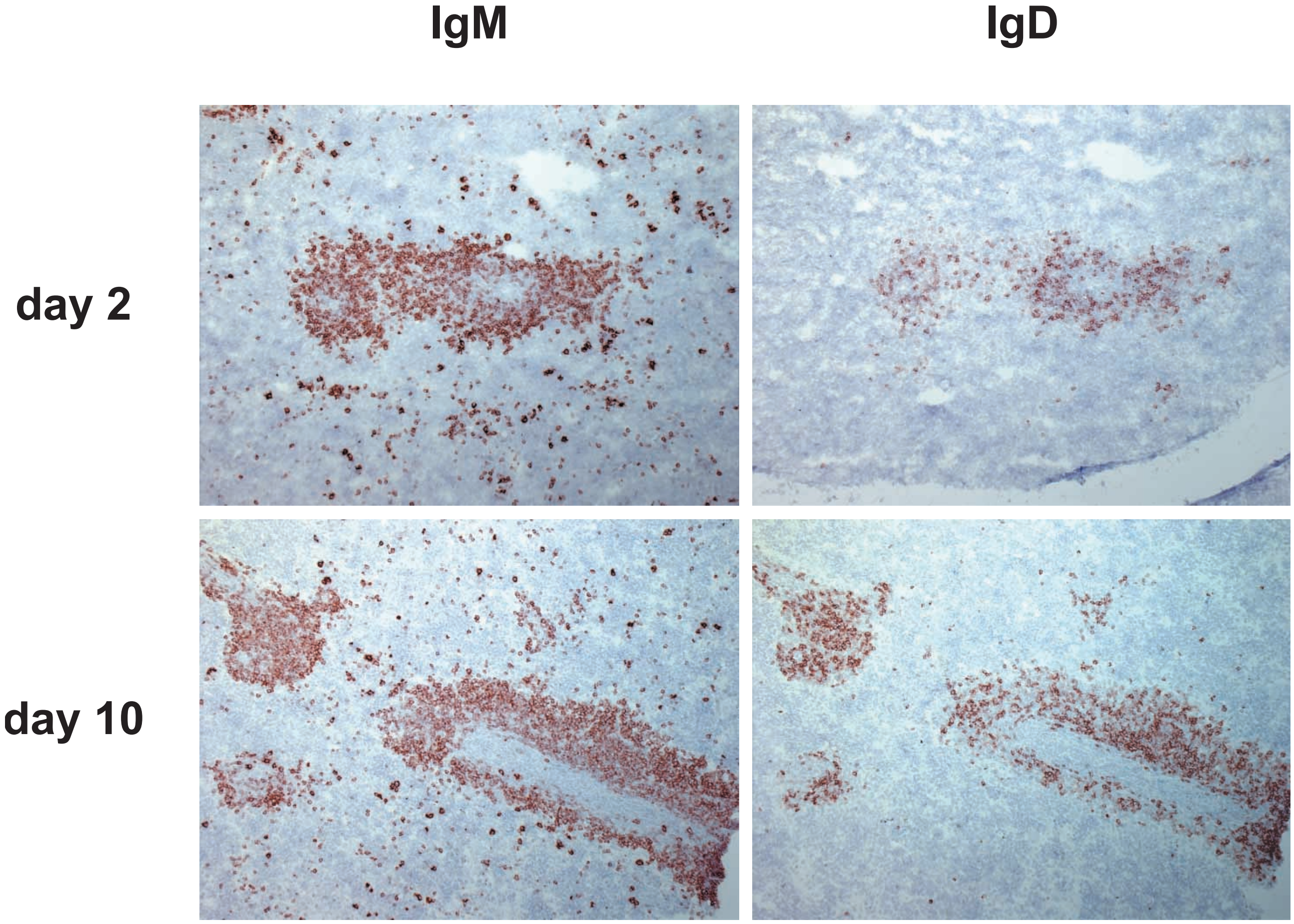

FIGURE 5 Indirect immunoperoxidase staining with anti-IgM (HIS40) and anti-IgD (MaRD3) of serial frozen sections of rat spleen 2 and 10 days after birth. At these time points, B cells are located in concentric areas surrounding the T cell areas (PALS); distinct B cell follicles and MZs are not present yet. Note that cells with the strongest membranous IgM staining are enriched at the outer zone of the concentric B cell areas, whereas IgD staining is confined to the inner parts.

follicle development is seen and a marginal sinus can sometimes be distinguished (Fig. 5). At this age, follicular type B cells ($IgM^+$, $IgD^+$ and $CD45R^+$) reside primarily in the inner parts of the concentric B cell areas. Cells in the outermost border of the concentric B cell areas display a strong membranous IgM and a weak HIS57 staining, but are $IgD^{low/neg}$ and $CD45R^{low/neg}$ (Fig. 5; data for HIS57 and CD45R not shown). The phenotype of these cells is typical for MZ-B cells. Thus, although follicles and MZ are not yet clearly developed in 2–10-days-old spleens, their characteristic B cells appear to be present already and somehow spatially separated from each other.

From 14 days after birth, B cell follicles and MZ are clearly recognized in spleen. Two-color indirect immunofluorescent staining (Fig. 6) of 14-days-old spleen shows that most B cells located in the MZ express high levels of HIS57 antigen and only low levels of CD45R (HIS24), and thus represent typical MZ-B cells. On the other hand, most B cells present in follicles exhibit strong CD45R (HIS24) staining and are virtually negative for HIS57 antigen. Therefore, these cells represent typical follicular type of B cells. Although most follicular B cells in both neonatal and adult rat spleen are $CD45R^{high}HIS57^{low/neg}$, Fig. 6 shows that a minority of the follicular B cells stain negative or weakly for CD45R (HIS24) but strongly for HIS57 antigen. These $CD45R^{low/neg}HIS57^{high}$ follicular B cells are predominantly confined to the outer zone of the follicles. We also observed that B cells in this region of the follicles frequently stain strongly for IgM and weakly for IgD (data not shown). Therefore, these follicular B cells resemble phenotypically MZ-B cells. We currently do not know whether these cells are MZ-B cells, immediate MZ-B precursor cells or represent a novel (follicular) B cell subset.

### DISCUSSION

Our study shows that already two days after birth, small numbers of MZ-B cells can be detected in rat spleen by flow cytometry. Similar to adult rats, these neonatal MZ-B cells are intermediate-sized $sIgM^{high}sIgD^{low}$ cells that express relatively low levels of CD45R (HIS24) and relatively high levels of HIS57 antigen. During the first 10 days after birth, however, immunohistologically defined MZ and follicles have not yet developed. In this period most splenic B cells reside in a distinct concentric area surrounding the PALS. Despite the lack of a morphologically distinct MZ, both MZ type and follicular type of B cells are present in neonatal spleen. MZ-B cells are enriched at the outermost border of the concentric areas, since cells located here exhibit strong membranous staining for IgM and are virtually negative for both IgD and CD45R (HIS24). Follicular B cells (ERF and RF B cells), on the other hand, are enriched at the inner part of the concentric areas, as the majority of cells at this location, stain for IgM, IgD and CD45R (HIS24). Our data therefore indicate that MZ-B cells appear earlier in ontogeny than initially described in the (immunohistological) study of Dijkstra and Dopp, 1983. In neonatal spleen, NF-B cells are located in the red pulp, as many cells in this area stain for IgM and CD45R (HIS24), but not for IgD. We cannot, however, rule out completely the possibility that (some) NF-B cells ($IgM^+IgD^-CD45R^+$) are also present in the concentric B cell areas.

Neonatal MZ-B cells display some subtle phenotypic differences with adult MZ-B cells. First, flow cytometry reveals that levels of HIS57 antigen on two-days-old MZ-B cells are about five times lower compared to adult rats. This lower expression level may explain the absence of HIS57 staining of spleen sections from two-days-old rats. Whether the lower HIS57 antigen expression levels have functional implications, awaits further characterization of the HIS57 antigen. Second, our study also shows that, in contrast to adult rats, neonatal MZ-B cells and RF-B cells express CD90 (Thy-1). CD90 is a heavily glycosylated, glycosyl phosphatidylinositol (GPI) anchored surface protein which is abundantly expressed on rodent thymocytes and mammalian neurons (Crawford and Barton, 1986; Firer *et al.*, 1995; Killeen, 1997). In adult rats, CD90 is also expressed on the surface of immature B cells (pro/pre-B, NF-B, and ERF-B), but is almost completely absent from RF-B and MZ-B cells. (Crawford and Goldschneider, 1980; Kroese *et al.*, 1995). Levels of CD90 on neonatal MZ-B cells diminish gradually with age; by three weeks after birth, these levels are still higher compared to adult MZ-B cells. The expression of this molecule on neonatal MZ-B cells may suggest that neonatal MZ-B cells are still immature. CD90 expression on neonatal RF-B and MZ-B cells is either due to active regulation of CD90 gene transcription or may reflect the fact that the vast majority of RF-B and MZ-B cells in neonatal animals are recently generated cells in which CD90 is not completely lost by turnover.

Timens *et al.*, 1989 and Zandvoort *et al.*, 2001a suggested that the lack of responsiveness to TI-2 antigens in early childhood (Pabst and Kreth, 1980; Rijkers *et al.*, 1998) is probably due to the immaturity of the splenic MZ-B cells in first two years of human life. This conclusion was based on their observation that splenic MZ-B cells of newborns lack, or weakly express, CD21 (CR2). We like to speculate here that the expression of CD90 on neonatal rat MZ-B cells might also have implications for their ability to respond to TI-2 antigens. Experiments with CD90-deficient ($Thy\text{-}1^{-/-}$) mice have demonstrated that CD90 negatively regulates (inhibits) TCR-mediated signaling in thymocytes (Hueber *et al.*, 1997). A similar inhibitory function of CD90 is also observed in neurons, where ligation of surface CD90 suppresses the outgrowth of neurites (Tiveron *et al.*, 1992). Thus, CD90 could also play an inhibitory role in B cell receptor (BCR) signaling and therefore negatively affect B cell responsiveness to TI-2 antigens.

In mice, MZ-B and B-1 cells share several phenotypic and functional characteristics, but differ, however, with respect to their developmental origin and anatomical location (Martin and Kearney, 2001). In contrast to splenic MZ-B cells, B-1 cells are self-replenishing and are

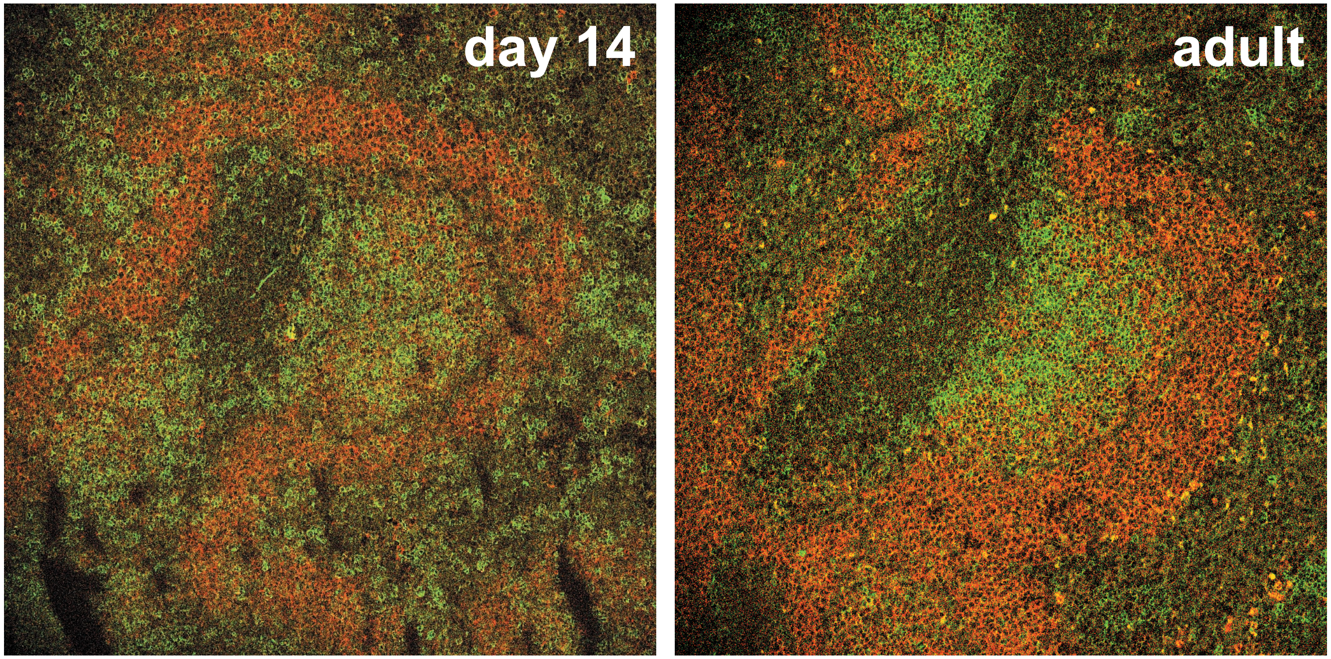

FIGURE 6 Two-color indirect immunofluorescent staining of frozen sections of the spleen from a 14-days-old neonatal rat (left) and a 5-months-old adult rat (right). Sections were stained for CD45R (HIS24; green) and HIS57 antigen (red). $CD45R^{neg}HIS57^{high}$ cells are enriched in outer zone of the follicles. Images were generated by confocal microscopy.

enriched in coelomic (peritoneal and pleural) cavities. There are no indications that MZ-B cells can become B-1 cells and *vice versa*. So far, we have not unequivocally detected the homologue of murine B-1 cells in the rat (Vermeer *et al.*, 1994). However, in a previous study, we observed a novel, previously unidentified B cell subset in the peritoneal cavity of SCID mice, reconstituted with rat fetal liver cells (rat → SCID mouse chimeras) (Deenen *et al.*, 1997). Flow cytometry analysis of these peritoneal rat-derived cells revealed the following phenotype: $sIgM^{high}CD45R^{low}HIS57^{high}$ $CD90^{int}$. We speculated that these cells might represent the rat homologue of murine B-1 cells. This study shows that neonatal MZ-B cells have the same phenotype. We consider it, however, as unlikely that the $sIgM^{high}CD45R^{low}HIS57^{high}CD90^{int}$ B cells in neonatal rat spleen are in fact, B-1 cells, because (i) CD90 expression levels on neonatal $sIgM^{high}CD45R^{low}$ $HIS57^{high}$ B cells gradually diminish with age and (ii) besides the $sIgM^{high}CD45R^{low}HIS57^{high}CD90^{int}$ cells, there are no substantial numbers of $sIgM^{high}CD45R^{low}$ $HIS57^{high}CD90^{neg}$ cells (i.e. the phenotype of adult MZ-B cells), even not at time points that MZs are well developed. Whether the peritoneal $sIgM^{high}CD45R^{low}$ $HIS57^{high}CD90^{int}$ B cells in rat → SCID mouse chimeras are indeed the rat homologue of murine B-1 cells still remains to be resolved.

### MATERIALS AND METHODS

### Animals and Flow Cytometry

Pregnant female and adult male PVG rats (PVG/OlaHsd) were obtained from Harlan Netherlands, Horst, The Netherlands. Male rats were obtained at 10 weeks of age and killed at 5-months of age. Rats were housed at the Central Animal Facility of the Faculty of Medical Sciences (University of Groningen, Groningen, The Netherlands) and reared under pathogen free (SPF) conditions. Spleen and bone marrow cell-suspensions were prepared as described before (Dammers *et al.*, 1999). Cells were stained for flow cytometry by a combination of the following FITC-conjugated, PE-conjugated or biotinylated mouse anti-rat mAbs: HIS40 (anti-IgM) (Kroese *et al.*, 1990), MaRD3 (anti-IgD; kind gift from Dr. H. Bazin, University of Louvain, Brussels, Belgium), HIS57 (Pharmingen, San Diego, CA) (Dammers *et al.*, 1999), OX-7 or HIS51 (CD90; Pharmingen), HIS24 (CD45R (B220); Pharmingen), and HIS50 (CD24, heat-stable antigen) (Hermans *et al.*, 1997). Biotinylated mAbs were revealed by streptavidin-allophycocyanin or streptavidin-Cy-Chrome (Pharmingen). FlowJo software (version 3.6; Tree Star, San Carlos, CA) was used for flow cytometry data analysis.

### Immunohistology of Splenic Tissue Sections

Splenic tissue was snap frozen in isopentane at −80°C and stored at −80°C. Indirect immunoperoxidase labeling of spleen sections was performed according to Zandvoort *et al.*, 2001b. The following mAbs were used for indirect immunoperoxidase labeling: HIS8 (Igκ), HIS24 (CD45R), HIS40 (IgM) (Kroese *et al.*, 1990), HIS57 (Dammers *et al.*, 1999), MaRD3 (IgD; kind gift from Dr. H. Bazin, University of Louvain, Brussels, Belgium), ER-04 (CD90) (Vaessen *et al.*, 1985), and R73 ($TCR_{\alpha\beta}$) (Hünig *et al.*, 1989). For indirect immunofluorescent labeling of spleen sections, cryostat sections of 5 μm were used. Sections were air-dried and fixed in acetone (−20°C) for 10 min. Slides were incubated for 1 h in phosphate buffered saline (PBS) containing appropriately diluted HIS24 (IgG2b) and HIS57 (IgG1) mAbs. After rinsing for 3 × 5 min with PBS, the slides were incubated for 30 min in PBS containing 3% (v/v) normal rat serum and appropriately diluted goat anti-mouse IgG2b (FITC) and goat anti-mouse IgG1 (TRITC) polyclonal antiserum (Southern Biotechnology Associates, Birmingham, AL). After rinsing for 3 × 5 min with PBS, sections were counter-stained using 4′, 6-diamidino-2-phenylindole, dihydrochloride (DAPI; Molecular Probes, Eugene, OR) according to the manufacturers recommendations, and subsequently embedded in Vectashield H-1000 mounting medium (Vector Laboratories, Burlingame, CA). Immunofluorescent staining was revealed on a Leica TCS SP2 Confocal Microscope using Leica imaging software (version 2.0; Leica Microsystems, Wetzlar, Germany).

### *Acknowledgements*

Authors thank Geert Mesander for flow cytometry assistance and Dick Huizinga for DTP.

### *References*

Benedict, C.L. and Kearney, J.F. (1999) "Increased junctional diversity in fetal B cells results in a loss of protective anti-phosphorylcholine antibodies in adult mice", *Immunity.* **10**, 607–617.

Crawford, J.M. and Goldschneider, I. (1980) "Thy1 antigen and B lymphocyte differentiation in the rat", *J. Immunol.* **124**, 969–976.

Crawford, J.M. and Barton, R.W. (1986) "Thy-1 glycoprotein: structure, distribution, and ontogeny", *Lab. Investig.* **54**, 122–135.

Dammers, P.M., de Boer, N.K., Deenen, G.J., Nieuwenhuis, P. and Kroese, F.G.M. (1999) "The origin of marginal zone B cells in the rat", *Eur. J. Immunol.* **29**, 1522–1531.

Dammers, P.M., Visser, A., Popa, E.R., Nieuwenhuis, P. and Kroese, F.G.M. (2000) "Most marginal zone B cells in rat express germline encoded Ig $V_H$ genes and are ligand selected", *J. Immunol.* **165**, 6156–6169.

Deenen, G.J., Dammers, P.M., de Boer, T. and Kroese, F.G.M. (1997) "Identification of a novel rat B cell subset in the peritoneal cavity of xenogeneic rat to mouse SCID chimeras", *Transplant. Proc.* **29**, 1752–1753.

Dijkstra, C.D. and Dopp, E.A. (1983) "Ontogenetic development of T- and B-lymphocytes and non-lymphoid cells in the white pulp of the rat spleen", *Cell Tissue Res.* **229**, 351–363.

Dunn-Walters, D.K., Isaacson, P.G. and Spencer, J. (1995) "Analysis of mutations in immunoglobulin heavy chain variable region genes of microdissected marginal zone (MGZ) B cells suggests that the MGZ of human spleen is a reservoir of memory B cells", *J. Exp. Med.* **182**, 559–566.

Feeney, A.J., Atkinson, M.J., Cowan, M.J., Escuro, G. and Lugo, G. (1996) "A defective $V_{kappa}$ A2 allele in Navajos which may play a role in increased susceptibility to *Haemophilus influenzae* type b disease", *J. Clin. Investig.* **97**, 2277–2282.

Firer, M.A., Zacharia, B.Z., Kostikov, M. and Irlin, Y. (1995) "The Thy-1 molecule: its properties and functions", *Isr. J. Med. Sci.* **31**, 382–386.

Friedberg, S.H. and Weissman, I.L. (1974) "Lymphoid tissue architecture. II. Ontogeny of peripheral T and B cells in mice: evidence against Peyer's patches as the site of generation of B cells", *J. Immunol.* **113**, 1477–1492.

Guo, W.X., Burger, A.M., Fischer, R.T., Sieckmann, D.G., Longo, D.L. and Kenny, J.J. (1997) "Sequence changes at the V-D junction of the $V_H$1 heavy chain of anti-phosphocholine antibodies alter binding to and protection against *Streptococcus pneumoniae*", *Int. Immunol.* **9**, 665–677.

Hermans, M.H., Deenen, G.J., de Boer, N., Bo, W., Kroese, F.G.M. and Opstelten, D. (1997) "Expression of HIS50 Ag: a rat homologue of mouse heat-stable antigen and human CD24 on B lymphoid cells in the rat", *Immunology* **90**, 14–22.

Hueber, A.O., Bernard, A.M., Battari, C.L., Marguet, D., Massol, P., Foa, C., Brun, N., Garcia, S., Stewart, C., Pierres, M. and He, H.T. (1997) "Thymocytes in Thy-$1^{-/-}$ mice show augmented TCR signaling and impaired differentiation", *Curr. Biol.* **7**, 705–708.

Hünig, T., Wallny, H.-J., Hartley, J.K., Lawetzky, A. and Tiefenthaler, G. (1989) "A monoclonal antibody to a constant determinant of the rat T cell antigen receptor that induces T cell activation. Differential reactivity with subsets of immature and mature T lymphocytes", *J. Exp. Med.* **169**, 73–86.

Killeen, N. (1997) "T-cell regulation: Thy-1-hiding in full view", *Curr. Biol.* **7**, R774–R777.

Kroese, F.G.M., Wubbena, A.S., Kuijpers, K.C. and Nieuwenhuis, P. (1987) "The ontogeny of germinal centre forming capacity of neonatal rat spleen", *Immunology* **60**, 597–602.

Kroese, F.G.M., Butcher, E.C., Lalor, P.A., Stall, A.M. and Herzenberg, L.A. (1990) "The rat B cell system: the anatomical localization of flow cytometry-defined B cell subpopulations", *Eur. J. Immunol.* **20**, 1527–1534.

Kroese, F.G.M., de Boer, N.K., de Boer, T., Nieuwenhuis, P., Kantor, A.B. and Deenen, G.J. (1995) "Identification and kinetics of two recently bone marrow-derived B cell populations in peripheral lymphoid tissues", *Cell. Immunol.* **162**, 185–193.

MacLennan, I.C.M. and Liu, Y.-J. (1991) "Marginal zone B cells respond to both polysaccharide antigens and protein antigens", *Res. Immunol.* **142**, 346–351.

Makowska, A., Faizunnessa, N.N., Anderson, P., Midtvedt, T. and Cardell, S. (1999) "CD1$^{high}$ B cells: a population of mixed origin", *Eur. J. Immunol.* **29**, 3285–3295.

Martin, F. and Kearney, J.F. (2000) "Positive selection from newly formed to marginal zone B cells depends on the rate of clonal production, CD19, and Btk", *Immunity* **12**, 39–49.

Martin, F. and Kearney, J.F. (2001) "B1 cells: similarities and differences with other B cell subsets", *Curr. Opin. Immunol.* **13**, 195–201.

Mond, J.J., Lees, A. and Snapper, C.M. (1995) "T cell-independent antigens type 2", *Annu. Rev. Immunol.* **13**, 655–692.

Mosier, D.E. and Subbarao, B. (1982) "Thymus-independent antigens: complexity of B-lymphocyte activation revealed", *Immunol. Today* **3**, 217–222.

Mosier, D.E., Mond, J.J. and Goldings, E.A. (1977) "The ontogeny of thymic independent antibody responses *in vitro* in normal mice and mice with an X-linked B cell defect", *J. Immunol.* **119**, 1874–1878.

Pabst, H.F. and Kreth, H.W. (1980) "Ontogeny of the immune response as a basis of childhood disease", *J. Pediatr.* **97**, 519–534.

Peltola, H., Kayhty, H., Sivonen, A. and Makela, H. (1977) "*Haemophilus influenzae* type b capsular polysaccharide vaccine in children: a double-blind field study of 100,000 vaccinees 3 months to 5 years of age in Finland", *Pediatrics* **60**, 730–737.

Rijkers, G.T., Sanders, E.A.M., Breukels, M.A. and Zegers, B.J.M. (1998) "Infant B cell responses to polysaccharide determinants", *Vaccine* **16**, 1396–1400.

Spencer, J., Perry, M.E. and Dunn-Walters, D.K. (1998) "Human marginal-zone B cells", *Immunol. Today* **19**, 421–426.

Tangye, S.G., Liu, Y.-J., Aversa, G., Phillips, J.H. and de Vries, J.E. (1998) "Identification of functional human splenic memory B cells by expression of CD148 and CD27", *J. Exp. Med.* **188**, 1691–1703.

Tierens, A., Delabie, J., Michiels, L., Vandenberghe, P. and de Wolf-Peters, C. (1999) "Marginal-zone B cells in the human lymph node and spleen show somatic hypermutations and display clonal expansion", *Blood* **93**, 226–234.

Timens, W., Boes, A., Rozeboom-Uiterwijk, T. and Poppema, S. (1989) "Immaturity of the human splenic marginal zone in infancy. Possible contribution to the deficient infant immune response", *J. Immunol.* **143**, 3200–3206.

Tiveron, M.C., Barboni, E., Pliego Rivero, F.B., Gormley, A.M., Seeley, P.J., Grosveld, F. and Morris, R. (1992) "Selective inhibition of neurite outgrowth on mature astrocytes by Thy-1 glycoprotein", *Nature* **355**, 745–748.

Vaessen, L.M., Joling, P., Tielen, F.J. and Rozing, J. (1985) "New surface antigens on cells in the early phase of T-cell differentiation, detected by monoclonal antibodies", *Adv. Exp. Med. Biol.* **186**, 251–260.

Vermeer, L.A., de Boer, N.K., Bucci, C., Bos, N.A., Kroese, F.G.M. and Alberti, S. (1994) "MRC OX19 recognizes the rat CD5 surface glycoprotein, but does not provide evidence for a population of CD5$^{bright}$ B cells", *Eur. J. Immunol.* **24**, 585–592.

Zandvoort, A., Lodewijk, M.E., de Boer, N.K., Dammers, P.M., Kroese, F.G.M. and Timens, W. (2001a) "CD27 expression in the human splenic marginal zone: the infant marginal zone is populated by naive B cells", *Tissue Antigens* **58**, 234–242.

Zandvoort, A., Lodewijk, M.E., Klok, P.A., Dammers, P.M., Kroese, F.G.M. and Timens, W. (2001b) "Slow recovery of follicular B cells and marginal zone B cells after chemotherapy: implications for humoral immunity", *Clin. Exp. Immunol.* **124**, 172–179.

